# Measuring the impact of sow farm outbreaks with PRRS virus on the downstream mortality using causal inference methods

**DOI:** 10.3389/fvets.2025.1545034

**Published:** 2025-04-30

**Authors:** E. S. Magalhães, D. Zhang, C. A. A. Moura, Annette O’Connor, C. Wang, D. J. Holtkamp, G. S. Silva, D. C. L. Linhares

**Affiliations:** ^1^Department of Animal Science, Iowa State University, Ames, IA, United States; ^2^Department of Statistics, College of Liberal Arts and Sciences, Iowa State University, Ames, IA, United States; ^3^Iowa Select Farms, Iowa Falls, IA, United States; ^4^Department of Large Animal Clinical Sciences, College of Veterinary Medicine, Michigan State University, East Lansing, MI, United States; ^5^Department of Veterinary Diagnostic and Production Animal Medicine, College of Veterinary Medicine, Iowa State University, Ames, IA, United States

**Keywords:** swine, breeding herd, PRRS outbreak, causal inference, nursery mortality

## Abstract

Porcine reproductive and respiratory syndrome virus (PRRSV) remains a significant challenge to the swine industry, resulting in substantial productivity and, consequently, economic losses. This study aimed to quantify the impact of PRRSV outbreaks in sow farms on nursery mortality using causal inference methods. The study design followed a retrospective observational approach, where PRRSV epidemic status in source sow farms was the exposure, and nursery mortality (percentage of dead pigs in the first 60 days post-weaning) was the outcome. Causal inference techniques were employed to estimate the effect of the exposure (PRRSV epidemic status) on the outcome (nursery mortality). Data from a Midwestern US swine production system comprising 2,592 lots of pigs, representing approximately 5 million pigs marketed between January 2021 and December 2022, were analyzed. A causal diagram was constructed to visualize the relationship between PRRSV epidemic exposure and nursery mortality, while controlling for potential confounding factors including season, average parity at farrow, and sow farm *Mycoplasma hyopneumoniae* status. Four analytical approaches were employed: univariate and multivariable regression models, propensity score matching, and a doubly robust method. The results indicated that PRRSV epidemic lots had higher nursery mortality compared to non-epidemic lots, regardless of the modeling approach used. The doubly robust method provided the most accurate estimates, offering lower mortality differences and narrower confidence intervals. This study demonstrated the application of causal inference methods on swine data to measure the impact of PRRSV on swine nursery mortality, which is an approach commonly used in other epidemiology areas but not well explored in veterinary epidemiology. The findings highlight the importance of employing causal inference models in veterinary epidemiology to improve the accuracy of disease impact assessments in field conditions, with potential applications in studying other pathogens or disease-related factors in livestock production.

## 1 Introduction

Porcine reproductive and respiratory syndrome virus (PRRSV) is one of the major pathogens in the global swine industry in terms of economic impact and productivity losses as it affects the health and performance across all pig ages and phases of production (1). Despite PRRSV being present in the U.S. since the late 1980s, the economic impact of PRRSV outbreaks in the U.S. was estimated to be approximately $664 million per year (2). In Europe, one study demonstrated an impact of €126 per breeding animal in Dutch sow farms (3).

The impact of PRRSV outbreaks in sow farms is well described in the literature, where multiple authors have described the reproductive impact characterized by increased number of abortions, pre-natal losses (i.e., stillbirth and mummified fetuses), and weak born suckling piglets, leading to increased pre-weaning mortality, and lower weaning weight (1). Similarly, PRRSV increases swine post-weaning mortality and reduces average daily weight gain.

When measuring the impact of PRRSV on the post-weaning growth performance, mortality is a key performance indicator (KPI) of swine operations. Mortality also relates to other performance indicators that allow to measure whether pig populations are reaching their genetic potential. However, swine mortality is a multifactorial problem. Thus, understanding webs of causal factors influencing post-weaning mortality requires applying a comprehensive analytical approach to reveal the role of any specific factor of interest on mortality.

Despite numerous studies focusing on swine pre-weaning mortality, fewer studies targeting the post-weaning causes of mortality have been reported (4). Multiple experimental and observational studies have been reported in a literature review on the impact of PRRSV on swine post-weaning mortality (2, 4–14). While traditional regression models are often used, causal inference methods such as doubly robust estimation may provide more accurate effect estimates under complex confounding structures.

Causal inference analysis overcomes the limitations when investigating such a complex outcome as swine mortality, as it addresses the potential confounding relationships using a causal diagram approach (15), and implements the correct adjustment of the background factors accordingly (16). One of the most common methods for controlling such confounders is based on propensity scores (17, 18).

Causal inference models applied to observational data are common in human medicine studies as some experimental studies may be difficult to conduct in humans (19). Despite the application of causal inference models in observational data not being a common practice in veterinary epidemiology (20), guidelines for conducting such approach in the veterinary epidemiology realm are available (16, 19, 21, 22) and conducted it to data collected under field conditions (23–25).

Therefore, the objective of this study was to describe the process of implementing causal inference concepts to swine data to measure the impact of sow farm PRRSV outbreaks on the downstream nursery mortality of cohorts weaned during the epidemic phase of infection and compare regression and causal inference methods to estimate PRRSV impact on mortality.

## 2 Materials and methods

### 2.1 Overview

This retrospective cohort study utilized data from 2,649 lots, representing approximately 5 million pigs, marketed between January 2021 and December 2022 from a midwestern US swine production system (Iowa Select Farms, Iowa Falls, IA). Breeding-to-market data were imported, cleaned, and integrated, creating a consolidated master table. After assessing the completeness of the data, lots with incomplete information were removed if information for any of the variables was missing. Productivity and health information was combined in the final master table for the remaining 2,592 eligible lots, utilizing a data management process previously described (41). The information available in the master table was analyzed implementing regression and causal inference methods to measure the impact of PRRSV outbreaks occurring in sow farms on the downstream nursery mortality of the progenies weaned within the first 16 weeks post-break.

### 2.2 Study design

This was a retrospective cohort study where the lots (*n* = 2,592) of pigs were the observational units. STROBE-Vet statement (46) for reporting observational studies was used to report the information in this manuscript. The observational unit was defined as one lot of pigs marketed from a growing site at one point in time (*n* = 2,592), where lots of pigs may have originated from a single or multiple sow farm(s). The exposure of interest was PRRSV epidemic infection in source sow farms. For the purpose of this study, an epidemic PRRSV status was defined as the 16 weeks after a reported PRRSV outbreak in breeding herds (12). The outcome was defined as the nursery mortality of the lot, expressed as the proportion of pigs that died during the first 60 days of the post-weaning phase, and calculated as a percentage of the total number of pigs placed. Lots were not included in this study when originating from multiple sow farms with different statuses for PRRSV or *Mycoplasma hyopneumoniae*. Lots with known lateral introduction of PRRSV in the growing phase (i.e., post-weaning phase) were not excluded from the analysis and were further considered as a variable in the model.

This study aimed to compare the nursery mortality (outcome) of pig lots weaned from PRRSV sow farms with two different PRRSV statuses (exposure), classified as epidemic or non-epidemic. Pre-weaning phase information consisted of productivity and health data from the sow farms, which was collected and integrated with the lots` closeout performance report, thus providing retrospective information from breeding-to-market for each of the 2,592 study pig lots.

A panel of five swine experts was formed within Iowa State University to discuss the information from the master table and to build a causal diagram (26, 27) illustrating the relationship between PRRSV status in sow farms and the nursery mortality, i.e., the exposure and outcome, respectively. Variables were selected based on biological plausibility, and factors identified as important but not available in the dataset were represented as unobserved in the diagram. Thereafter, the variables identified as potential confounders in the causal diagram were then utilized in five different analytical approaches to estimate the effect of the exposure on the outcome.

### 2.3 Data characteristics

The following definitions describe the variables involved in the relationship between the effect of PRRSV outbreak in sow farms on the downstream nursery mortality of the weaned lots of pigs. The data utilized as the exposure, outcome, and the remaining variables controlled in this relationship are provided in Table 1.

**Table 1:** Description of the variables demonstrated on the causal diagram.

Variable name	Classification	Type	Category	Description
Nursery mortality	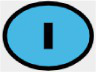 Outcome	Continuous	Continuous variable	Proportion of pigs dead based on the pigs placed in
PRRSV status	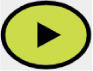 Exposure	Categorical	Epidemic	Pig lot classification according to sow farm PRRSV status at weaning
			Non-epidemic	
*M. hyopneumoniae* status	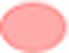 Confounder	Categorical	Endemic	Lots` classification according to sow farm *M. hyo* status at weaning
			Negative	
Average parity at farrow^a^	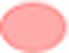 Confounder	Categorical	3.0 farrows	Average litter parity of the lots at weaning
			3.8 farrows	
			4.2 farrows	
			4.9 farrows	
Season	Confounder 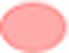	Categorical	January–March	Months when pigs were weaned: January–March (1st) April–June (2nd) July–September (3rd) October–December (4th)
			April–June	
			July–September	
			October–December	
Sow source	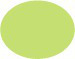 Ancestor of exposure	Discrete	47 sow farms	Sow farm originating the lots of pigs.
PRRSV nursery outbreak	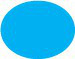 Ancestor of outcome	Categorical	PRRSV break	Classification according to PRRSV outbreak in the post-weaning phase
			No break	
Stocking weight	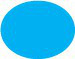 Mediator	Continuous	~15 lbs.	Average stocking weight of the lots at placement
Pre-wean mortality	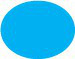 Mediator	Continuous	~14%	Average pre-weaning mortality of the lots
Average weaning age	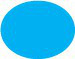 Mediator	Continuous	~20 days	Average stocking weight of the lots at placement
Other diseases	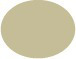 Unobserved	–	–	Other diseases occurring in the pre-weaning phase

* 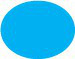 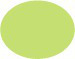 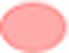 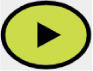 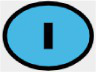 Type of the variable.

a Average number of farrows per group category.

#### 2.3.1 Exposure variable

The pig lots were classified into two observational categories based on their PRRSV status at the time of weaning from sow farms: “Epidemic” for pigs lots weaned during the first 16 weeks of a PRRSV outbreak in the sow farm source and “non-Epidemic” for those weaned after that period (≥ 17 weeks).

#### 2.3.2 Outcome variable

Nursery mortality was the outcome of interest in this study and represented the percentage of dead pigs during the initial 60 days of the post-weaning phase for each pig lot. A Shapiro–Wilk test, through a univariate analysis, was conducted to verify if the distribution of the outcome was normalized, and the log-transformed nursery mortality was used in further analysis.

#### 2.3.3 Conditional variables

The other variables involved in the relationship between the aforementioned exposure and outcome were proposed through the causal diagram and were classified as either ancestors of exposure, ancestor of outcome, or ancestor of both (Table 1). Variables identified as ancestor of both exposure and outcome simultaneously refer to confounders (28), which are factors that need to be adjusted to estimate the correct total effect of the exposure on the outcome.

### 2.4 Causal diagram

**Figure 1:**
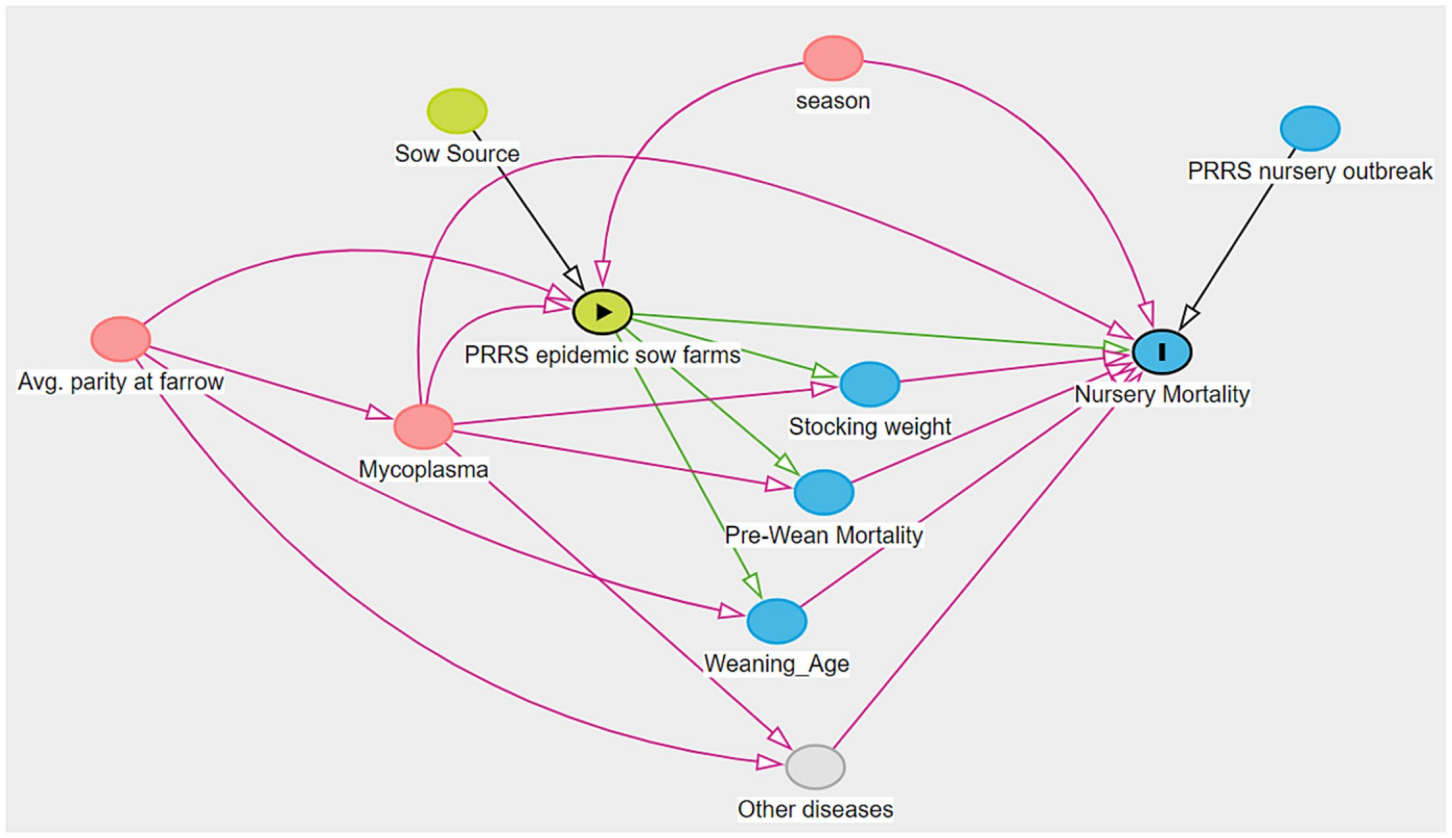
Causal diagram for the sow farm PRRSV status impact of nursery mortality. * The minimum sufficient set (MSS) of variables to measure the total effect of PRRS epidemic sow farms vs. non-epidemic sow farms included three confounders: average parity at farrow, *Mycoplasma hyopneumoniae* status, and season. The exposure is represented by 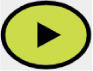, while 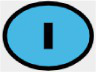 is the symbol for the outcome nursery mortality, and factors are represented by 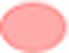 are denoted as confounders, as they cause the exposure and outcome simultaneously. The pathways represented by 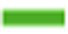
indicates a causal pathway, while 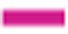
is a potential biasing pathway that needs to be blocked.

Prior to conducting the statistical analyses to measure the effect of PRRSV outbreaks on nursery mortality, a causal diagram was made by a panel of five experts at Iowa State University and Iowa Select Farms to illustrate and inform which factors are involved in the possible causal relationships between the impact of PRRSV epidemic flows from sow farms on the downstream nursery mortality of weaned lots. For this purpose, the DAGitty web interface (29) was utilized to:

(1) Create the visual representation of the direct acyclic graph (DAG) represented in Figure 1, where the possible pathways between the exposure and outcome were illustrated.
(2) Assess the potential direct and total effect of the exposure on the outcome.
(3) Identify the minimum sufficient set to be adjusted before measuring the total effect of PRRSV on nursery mortality.

On the causal diagram (Figure 1), the exposure is represented by the 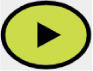 symbol and refers to sow farms facing during the epidemic phase of a PRRSV outbreak. The symbol for the outcome nursery mortality is 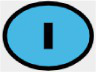, and factors are represented by 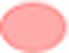 which are denoted as confounders as they cause the exposure and outcome simultaneously. The pathways represented by 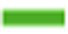 indicate a causal pathway, while 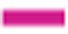 is a potential biasing pathway (backdoor) that needs to be blocked.

The direct effect of exposure on the outcome is represented by a single arrow linking PRRSV epidemic flows (exposure) directly to downstream nursery mortality (outcome). All other indirect arrows intermediating the pathways between exposure and outcome are denoted as indirect paths, where mediator variables were present. As mentioned above, the total effect and direct effect refer to the overall effect of the exposure accounting for all mediators, and the effect refers to the remaining impact without the mediators.

The DAG constructed in this study identified the following variables as potential confounders in the relationship between PRRSV epidemic lots and nursery mortality: season (time of the year when lots were weaned), average parity at farrow (average parity of the sows that farrowed and weaned piglets), and sow farm *Mycoplasma hyopneumoniae* status (farms classified as either endemic or negative). The lateral introduction of PRRSV in the nursery phase was also considered an important variable, here referred as PRRSV nursery outbreak, that was controlled in the regression analysis approaches but was not controlled as a confounder in the causal approaches described below, as it was not present in the causal pathway between exposure and outcome (25, 30), but affects the outcome and the estimation of PRRSV impact in the post-weaning phase.

### 2.5 Statistical analyses

Four analytical approaches were used to estimate the effect of PRRSV status on nursery mortality: two based on regression models (outcome modeling) and two based on propensity score methods (exposure modeling). The outcome models included a univariate and a multivariable regression model, where nursery mortality (log-transformed) was regressed of PRRSV status. The other two causal models involved were based on propensity scores (18), where one matching, and the other applied a doubly robust approach that combined exposure and outcome modeling. The variables identified as potential confounders on the causal diagram are used in both outcome and exposure modeling approaches as either a covariate or a matching variable, respectively. The results reported in each approach referred to significance (*p* < 0.05), mean nursery mortality, and mean confidence interval (C.I.) for each exposure category.

#### 2.5.1 Outcome models

On the outcome model, two methods were utilized to estimate the exposure’s effect on the outcome using PROC GLIMMIX on SAS^®^ Version 9.4 (SAS Institute, Inc., Cary, NC). The first refers to a univariate model (UM), where the numerical outcome, log-transformed nursery mortality, is regressed on the binary exposure, and the differences in nursery mortality are measured in the two categories of the exposure (PRRSV epidemic vs. non-epidemic), without any covariate in the model (ANOVA). Similarly, the second approach is a multivariable model (MM) where the log-transformed nursery mortality is regressed on the exposure, along with a set of covariates (i.e., all confounders and PRRSV nursery outbreak as mentioned above) selected based on the causal diagram to be controlled.

#### 2.5.2 Exposure models

For the exposure model, two other models were utilized to measure the effect of the exposure on the outcome by utilizing propensity score causal methods. The first approach consisted of a propensity score model (PS) using PSMATCH procedure on SAS, where a matched dataset was built based on the propensity score values of each observation. The propensity score methodology was first described by Rosenbaum and Rubin (18) and refers to the probability of each observation being exposed given a set of covariates. This represented the probability for each group in the study population being either PRRSV epidemic or non-epidemic, given the confounders identified in the causal diagram.

**Figure 2:**
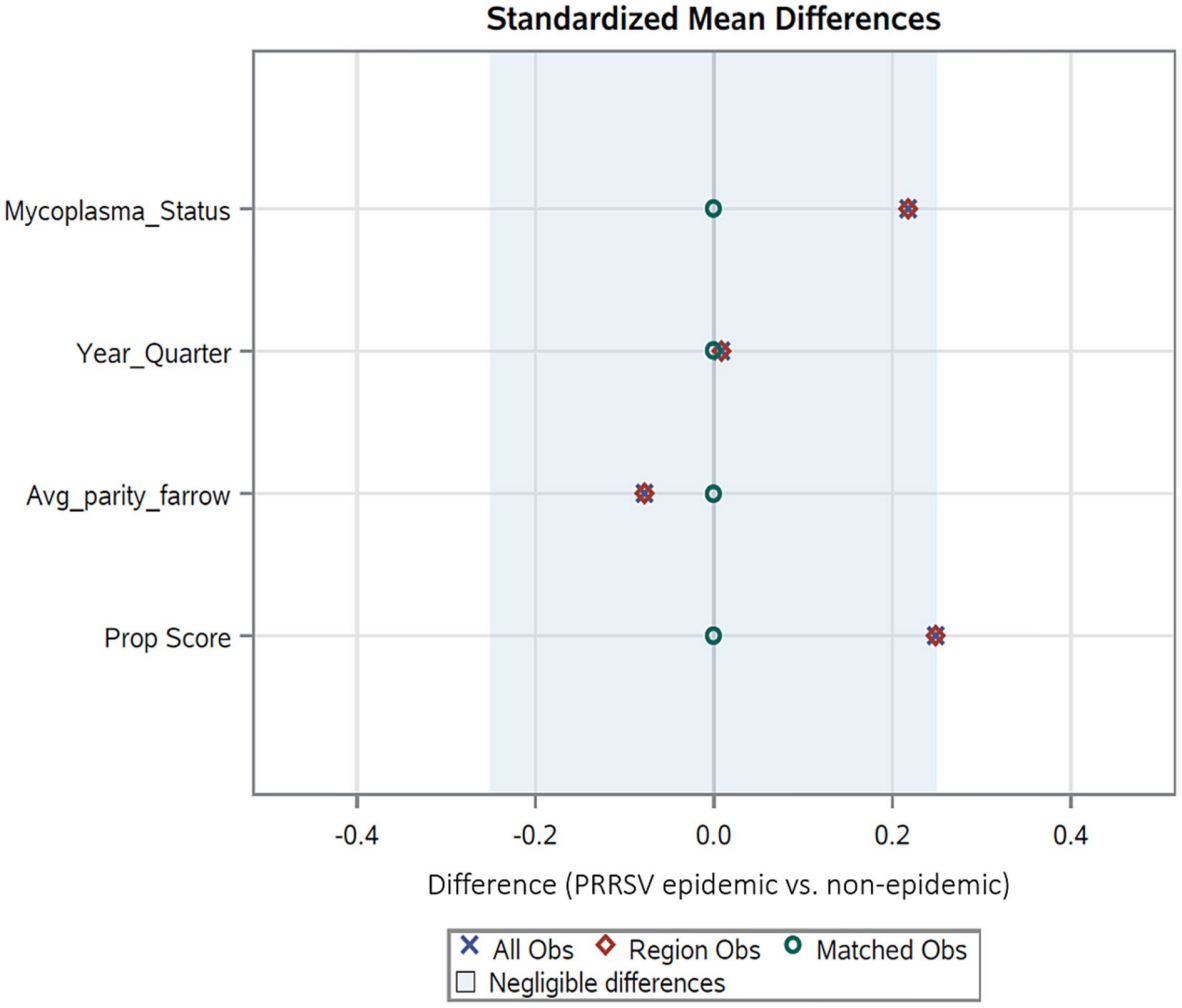
Standardized mean differences of the covariates after matching with PSMATCH. All observations and region observations refer to the complete dataset and the region utilized to find matches, respectively.

The matched dataset was created by selecting one PRRSV non-epidemic pig lot for each lot classified as PRRSV epidemic, based on approximate propensity score value (i.e., similar background confounding effects). After assessing if the propensity score differences were reduced before and after creating a matched dataset (Figure 2), the causal effect of lots originating from PRRSV epidemic sow farms on downstream nursery mortality was estimated compared to the non-epidemic lots by using a paired *t*-test. The matching method using propensity score measures only the average treatment effect of the treated, often referred as ATT (31), which represents the effects of the exposure within the exposed population.

The second modeling approach utilized in this study is the doubly robust method using CAUSALTRT on SAS, which combined an exposure model based on the inverse probability weighting (IPW) method (32) and an outcome model based on regression adjustment estimation. The IPW utilizes a logistic regression model that regresses the binary exposure (PRRSV epidemic vs. non-epidemic) on the confounders, to estimate the propensity scores of each group in the dataset, and the inverse of these values is used to weight each individual in the estimation of the outcome.

Combining the two modeling approaches in a single model is called doubly robust because it produces an unbiased estimation of the causal treatment effect even if there are misspecifications on the models (33, 34), as long as one of the models is correctly specified. The causal effect estimated using this approach is defined as the average treatment effect (ATE), which refers to the effect of the exposure within the entire study population, meaning, for this study, the effect of the exposure PRRSV epidemic flows for epidemic and non-epidemic lots (31).

## 3 Results

### 3.1 Data characteristics

This study was initiated with information from 2,649 lots, where 57 lots were deleted due to incomplete information for the variables across all observational units. The final data prepared for analyses contained 2,592 lots marketed from January 2021 until April 2023. A total of 188 lots were classified as PRRSV epidemic concerning their historical status at the time of the weaning from sow farms and the remaining lots (*n* = 2,068) as non-epidemic. Lots within the non-epidemic category included sow farms after the 16-week period post-outbreak, and positive stable sow farms, which is equivalent to categories I-B, II, and II-vx described in a recent PRRSV classification system (35).

The Shapiro–Wilk test on the outcome demonstrated that distribution of nursery mortality was not normal for the study population, thus requiring its log-transformation before the analyses. The back-transformed geometric mean nursery mortality was 3.42% (95% confidence interval (CI) 3.32–3.53%). The descriptive results of the variables in this study according to the exposure status (PRRSV epidemic vs. non-epidemic) are demonstrated in Table 1.

### 3.2 Causal diagram

The relationship among all variables included in this study is presented as a DAG in Figure 1. Between exposure (PRRSV epidemic sow farms) and outcome (nursery mortality), there were eight factors involved in this relationship, and one represents a post-weaning event interfering with the outcome only (i.e., not related to exposure → outcome directionality).

The variables identified with a blue circle refer to mediators as they are ancestors of the outcome but are influenced by the exposure status. On the other hand, the directionality of the variables in red simultaneously impacts both outcome and exposure. Blue and red variables are referred in the literature as mediators and confounders, respectively (30, 36).

The variable “other diseases” in gray is also a mediator but was considered unobserved as no data were available regarding other diseases occurring in the sow farms. Similarly, “Sow Source” represents sow farm-related factors that may influence the impact of PRRSV infection on pig health and mortality. Finally, as mentioned previously, “PRRSV nursery outbreaks” was a factor identified on the DAG that influences the outcome directly, but it is not related to exposure → outcome directionality as it concerns a post-weaning factor. For this reason, groups with a PRRSV outbreak during the nursery phase were adjusted in the regression analyses and not in the causal inference models.

As it was not our goal to estimate the direct effect, the minimal sufficient set (MSS) to correctly measure the total causal effect of sow farm PRRSV epidemic vs. non-epidemic groups on nursery mortality included three confounders: Average parity at farrow, season, and *Mycoplasma hyopneumoniae.* Table 2 contains the descriptive values for group frequency and nursery mortality means for each confounder for the total effect, according to the exposure status (epidemic vs. non-epidemic).

**Table 2:** Description of the variables indicated to be controlled on the causal diagram.

Variable name	Categories	Nursery mortality^a^	Groups` mortality (%) and frequency (*n*)	
			PRRSV epidemic	PRRSV non-epidemic
*Mycoplasma hyopneumoniae* status	Endemic	5.39%	12.10% (*n* = 135)	4.78% (*n* = 1,481)
	Negative	3.61%	7.41% (*n* = 53)	3.39% (*n* = 923)
Average parity at farrow	3.08	5.16%	10.89% (*n* = 51)	4.69% (*n* = 612)
	3.84	4.87%	10.71% (*n* = 53)	4.34% (*n* = 580)
	4.24	4.34%	11.18% (*n* = 41)	3.88% (*n* = 605)
	4.92	4.49%	10.34% (*n* = 43)	3.78% (*n* = 607)
Season	January–March	5.82%	11.60% (*n* = 63)	5.10% (*n* = 505)
	April–June	4.39%	9.36% (*n* = 27)	4.18% (*n* = 620)
	July–September	3.82%	9.60% (*n* = 33)	3.58% (*n* = 798)
	October–December	5.33%	11.17% (*n* = 65)	4.54% (*n* = 481)

a Raw mean nursery mortality percentage for each variable and the respective categories.

### 3.3 Measuring the total effect of PRRSV epidemic groups

The results from the four approaches tested in this research concerning the nursery mortality of lots classified as either PRRSV epidemic or non-epidemic are demonstrated in Table 3.

**Table 3:** Results of the multiple approaches for measuring the effect of exposure on outcome.

Type of model	Sow farm PRRSV status	Nursery mortality^a^	Confidence interval (C.I.)	*p*-value
Univariate Model (UM)	Epidemic	8.43%	7.59–9.37%	<0.0001
	Non-epidemic	3.19%	3.09–3.28%	
Multivariable Model (MM)	Epidemic	7.49%	6.70–8.36%	<0.0001
	Non-epidemic	3.10%	2.99–3.23%	
Matched Dataset (PSMATCH)	Epidemic	8.43%	7.59–9.37%	<0.0001
	Non-epidemic	3.64%	3.12–3.88%	
Doubly robust (DR)	Epidemic	7.18%	6.45–7.98%	<0.0001
	Non-epidemic	3.21%	3.12–3.31%	

a Geometric mean nursery mortality for each variable and the respective categories, back transformed from the log-scale. The controlled variables are average parity at farrow, *Mycoplasma hyopneumoniae* status, and season.

The outcome model approaches were based on regression methods only, and propensity scores were not utilized. The univariate model had the highest nursery mortality for the PRRSV epidemic category compared to the remaining approaches and the largest difference between epidemic and non-epidemic lots. Notably, this approach did not control for any confounders by including covariates and simulated the comparison of raw means, or similar to a “pivot-table” approach. On the other hand, the multivariable model had lower estimates and a narrower confidence interval compared to the univariate model.

For the exposure model approach, which is based on propensity scores for reducing variation between the confounders indicated by the causal diagram, the matching approach (PSMATCH) resulted in selecting 188 non-epidemic lots with similar propensity scores to the 188 epidemic lots. Using this matched dataset (*n* = 376 lots), the geometric mean nursery mortality for the epidemic lots was identical (8.43%) to the univariate approach discussed above, but the non-epidemic lots` estimate was higher (3.64%). Figure 2 demonstrates that the standardized mean differences for the covariates reduced to near zero when using the matched dataset.

The last model utilized in the exposure model approach was the doubly robust method, which combined modeling the exposure variable, here using the inverse probability weighting (IPW), and modeling the outcome with the regression method used on multivariate model. The method provided an estimate of the mean nursery mortality differences between PRRSV epidemic and non-epidemic lots for the entire population (ATE).

**Figure 3:**
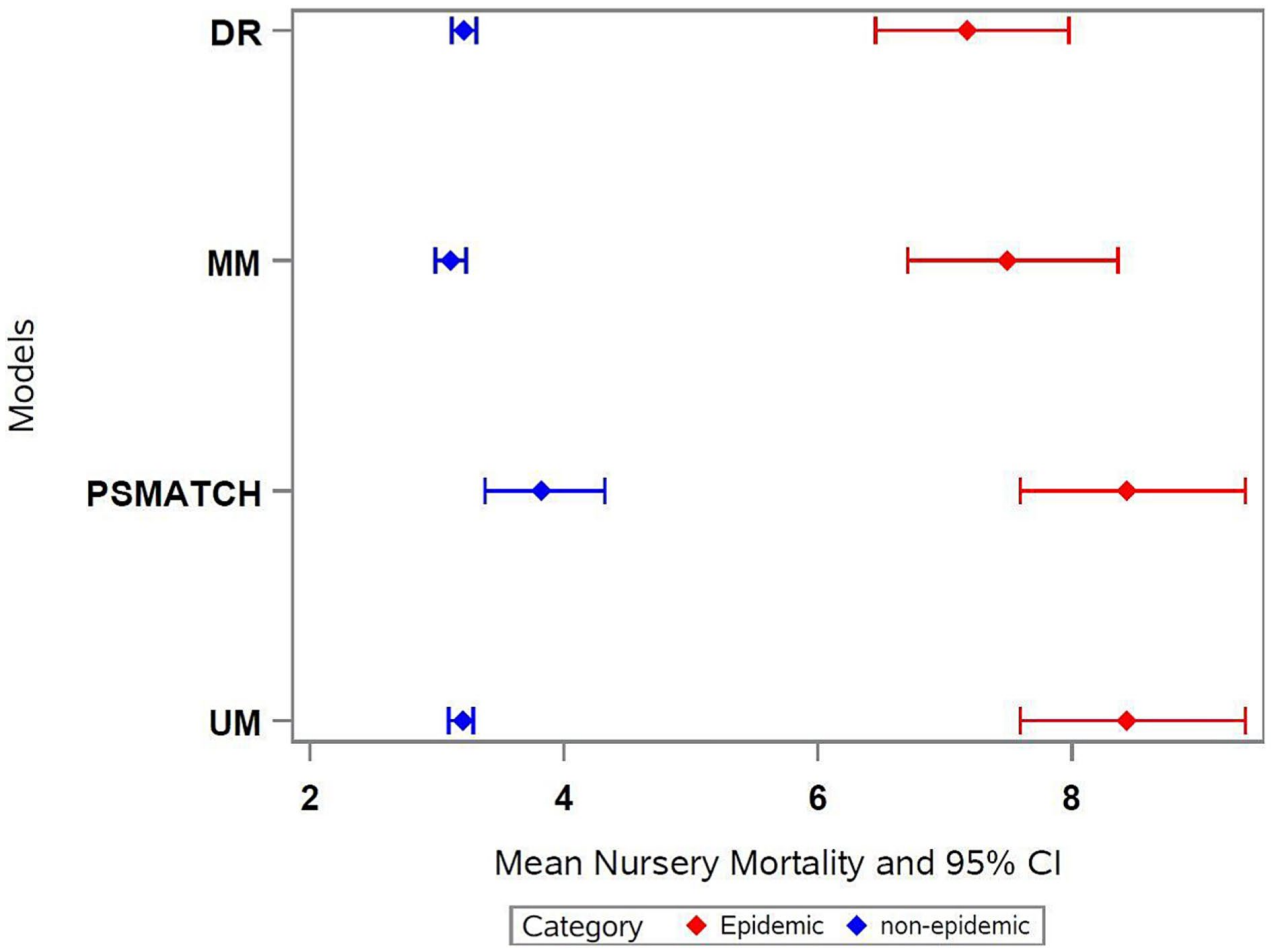
Estimates and C.I. for different approaches on measuring PRRSV status impact. DR, doubly robust; MM, multivariable model; PSMATCH, matching method using propensity score; UM, univariate model.

**Figure 4:**
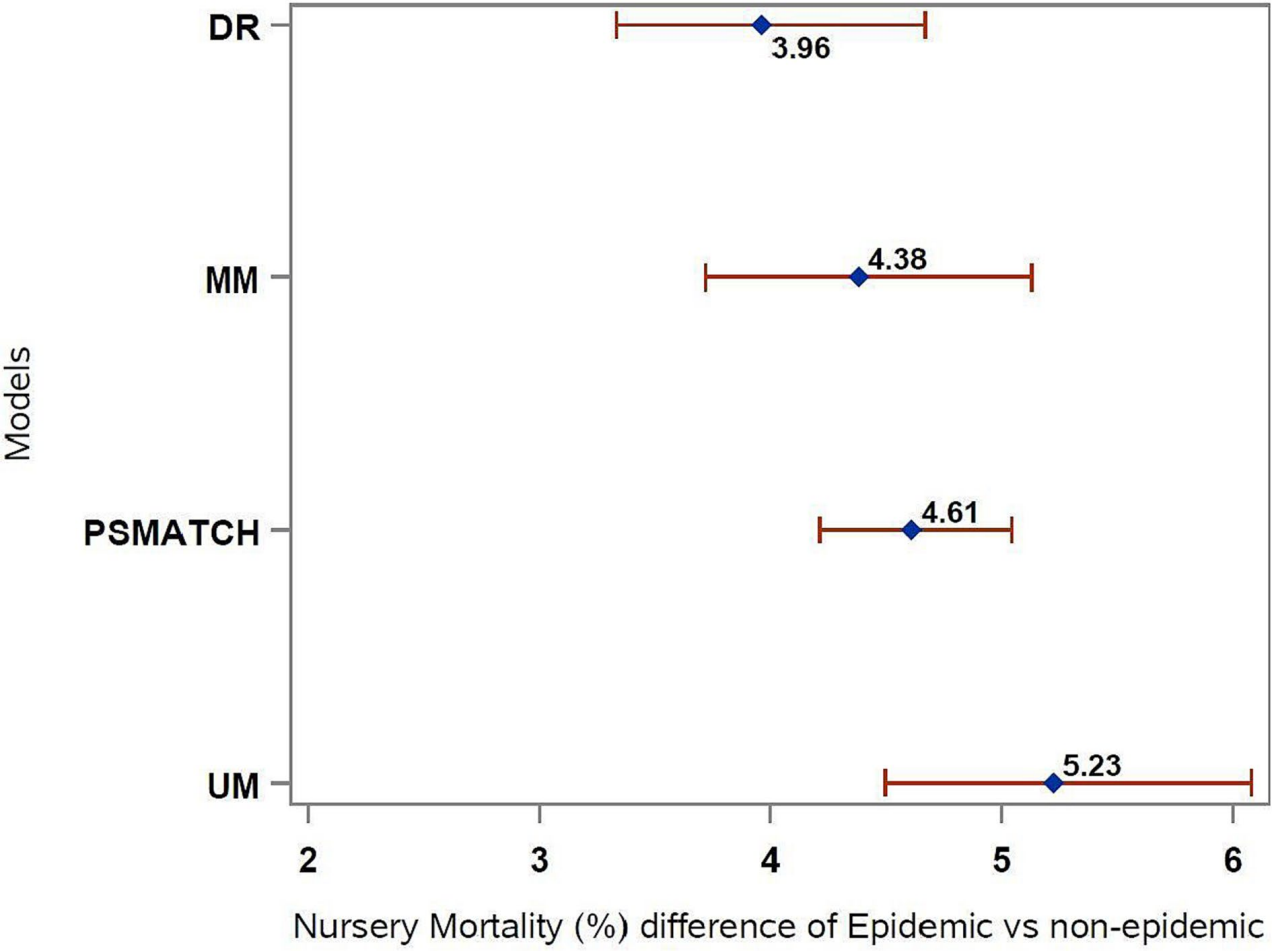
Difference in mortality between epidemic and non-epidemic groups for different approaches. DR, doubly robust; MM, multivariable model; PSMATCH, matching method using propensity score; UM, univariate model.

This method had the lowest estimates of mortality for both categories compared to the other three approaches. Similarly, narrower confidence intervals for the estimates were obtained, as well as a smaller difference in nursery mortality when comparing epidemic versus non-epidemic lots (Figures 3, 4).

When examining covariate balance (Table 4) based on the propensity score values calculated, the DR model reduced the majority of the variation between the covariates after weighting. This was observed based on reduced standardized mean differences between epidemic and non-epidemic (values closer to 0) and variance ratio values (values closer to 1). The variable average parity at farrow was the factor with less reduction across the categories, where one (4.24 category) had an increased difference after weighting. The blank values observed were not calculated as they refer to each covariate’s reference values.

**Table 4:** Covariate differences for propensity score on the doubly robust (DR) model.

Variable name	Categories	Standard difference^a^		Variance ratio^b^	
		Unweighted	Weighted	Unweighted	Weighted
*Mycoplasma hyopneumoniae* status	Endemic	0.2178	0.0311	0.8559	0.9833
	Negative	–	–	–	–
Average parity at farrow	3.08	0.0379	0.0321	1.0417	1.0352
	3.84	0.0926	−0.0391	1.1059	0.9525
	4.24	−0.0793	−0.0904	0.9055	0.8903
	4.92	–	–	–	–
Season	January–March	0.2836	0.0100	1.3427	1.0135
	April–June	−0.2883	−0.0355	0.6426	0.9581
	July–September	−0.3654	0.0147	0.6526	1.0111
	October–December	–	–	–	–

a Standard difference: values closer to zero on the weighted data vs. unweighted data mean less difference between epidemic and non-epidemic exposure lots.

b Refer to the ratio of the variance to the mean, where values closer to 1 also indicated less variation between exposure lots.

## 4 Discussion

In this study, we aimed to measure the causal effect of sow farm PRRSV outbreaks on the nursery mortality of pig lots weaned within the epidemic and non-epidemic periods, utilizing data collected under field conditions and comparing analytical approaches based on regression or causal inference methods.

When comparing the modeling approaches for estimating the effect of PRRSV, we observed increased nursery mortality for lots of pigs originating from PRRSV epidemic sow farms versus non-epidemic, which was statistically significant across all methods tested. The non-epidemic sow farms included herds not within the first 16 weeks of an outbreak, which represented unstable low prevalence or stable sow farms, similar to category I-B, II, and II-vx described in a recent PRRSV classification system (35). The estimates for the univariate model, which does not control for any confounding factors, demonstrated increased nursery mortality of 5.23% in PRRSV epidemic lots compared to non-epidemic (8.43% vs. 3.19%, respectively). PSMATCH estimates for PRRSV epidemic lots were the same as the UM estimates (8.43%), but the marginal estimates for non-epidemic lots were higher (3.64%), resulting in a smaller difference (4.69%) compared to UM (5.23%).

The smaller mortality estimates for PRRSV epidemic lots were observed on the doubly robust approach (7.18%), which was 3.97% higher compared to PRRSV non-epidemic lots (3.21%). Similarly, the results from the multivariate model (MM) were more similar to the DR model, with nursery mortality estimates for the PRRSV epidemic 4.38% larger than the nursery mortality for non-epidemic lots (7.48% vs. 3.10%, respectively).

The results of this study indicate a difference in the distribution/frequency of the background factors listed through the causal diagram before and after applying any of the methods to control confounding effects. Thus, estimating the effect of PRRSV sow farm outbreaks on the downstream nursery mortality without any adjustment for confounders (i.e., using the UM approach) is imprecise and can lead to biased results (17).

The PSMATCH result for non-epidemic lots compared to the univariate model is a consequence of matching non-epidemic lots with similar characteristics to epidemic cohorts based on similar propensity scores, given the confounding factors. Thus, selecting PRRSV non-epidemic lots with similar conditions for the confounders likely selected lots challenged with other diseases not related to PRRSV outbreaks, for example, non-epidemic lots during winter months, a season with higher detection of swine enteric and respiratory pathogens (13, 37).

The doubly robust estimation of increased nursery mortality of 3.97% in PRRSV epidemic flows compared to non-epidemic lots suggested the most accurate estimate for this study population, as seen that standard mean differences for the confounders after weighting decreased, and the lowest confidence intervals were observed (i.e., lower errors) compared to the remaining modeling approaches. As the true causal impact of PRRSV in nursery mortality is impossible to measure, it is theoretically favorable to utilize the doubly robust estimation methods for causal inference analysis in veterinary epidemiology, as it combines the outcome regression model with a model for the exposure based on the propensity score, thus providing unbiased results as long as one of the two models are correctly specified (34).

The range of weeks selected in this study to classify batches of weaned pigs as PRRSV epidemic (first 16 weeks of an outbreaks) and non-epidemic does not exclude the possibility of having non-epidemic groups with clinical signs similar to the epidemic batches of weaned pigs, mixed with groups without clinical signs. Previous authors reported breeding herd outbreaks taking longer than 17 weeks to “cool down” the effect of PRRS associated with PRRSV shedding and clinical signs (13, 14, 38). Therefore, the results of the present study could be underestimated if the acute phase of infection is occurring in both classifications hereby utilized. For instance, an industry report that analyzed 49 breeding herds with PRRSV outbreaks demonstrated median time to baseline production (i.e., time to reach productivity regarding the number of pigs weaned before the outbreak) was 22 weeks (39). On the other hand, this industry report showed that the largest productivity losses in terms of number of weaned pigs were higher during the initial 16 weeks but could have lasted longer periods.

The results of this research are in accordance with other veterinary studies that observed a more precise estimation of marginal effects when investigating the causal inference of an exposure using the doubly robust methods compared to regressions models (24, 25). Notably, regression models should provide similar results to the propensity score-based methods if no confounding is present or if the relationship between the variables is linear (23). However, complex non-linear relationships between many extraneous variables related to the exposure and outcome being investigated are common in the veterinary epidemiology realm, where propensity score can be a good solution for epidemiologists as it demonstrates higher modeling robustness (40). In addition, potential residual confounding due to the inclusion of nodes that are proxy of other confounders can occur, for example, with season, as it is a proxy of periods of the year of lower or higher incidence of disease.

The clinical presentation of PRRSV in swine herds may vary from subclinical to devastating, depending on multiple concurrent factors (1). Previous observational studies in the literature have reported PRRSV impact incoming from sow farms on the downstream mortality by increasing post-weaning mortality by 1.78% (5), 3.4% (14), 3.3% (2), and up to 10.19% (41), but in these cases sow farms classified as positive for PRRSV included cohorts with positive detection of virus through rt-PCR in due-to-wean age piglets, which may last over 25 to 32 weeks after an outbreak (38). On the other hand, the devastating effect was demonstrated in case studies available from the literature reporting outbreaks in sow farms of high pathogenicity PRRSV, which caused peaks in downstream wean-to-finish mortality reaching ~20–50% (6, 11, 42), and nursery mortality averaging 9.9% for the batches weaned within the first 18 weeks after an outbreak (9).

Despite PRRSV being one of the most relevant diseases for the global swine industry (1–3), and common within the United States (43–45), research on its impact under field conditions is limited to observational studies as mentioned above. In these cases, adjustment for extraneous variables was done through regression models and without prior identification of confounders through a causal diagram. Notably, no prior observational studies have measured the impact of diseases in swine using causal inference methods, and, to the best of the author’s knowledge, this is the first observational study measuring the causal impact of PRRSV on swine nursery mortality.

Unfortunately, the scenario of limited causal inference research is not different for other livestock species. In fact, a review of two hundred observational studies (20) in veterinary medicine conducted between 2020 and 2022 reported that 86% of the scientific studies utilized the causal wording incorrectly (i.e., in the absence of a clearly defined exposure and outcome relationship, and without methods for controlling for confounders or a causal diagram). Only one study out of two hundred reported explicit exposure and outcome relationships, along with a causal diagram.

The absence of research applying causal inference methods for measuring PRRSV’s impact is not due to the lack of information related to its extraneous variables (i.e., other risk factors) as disease-related information is commonly recorded under field conditions (19). Actually, the availability of information concerning confounding factors for the lots of pigs in this study was what made it possible to build the causal pathway (DAG), which guided the subsequent statistical analyses without requiring to record extra information. Furthermore, plenty of information is available in the literature concerning the guidelines for conducting causal inference analysis from observational data in the veterinary epidemiology realm (16, 22).

The external validity of the present study is limited to the swine production system from which the data were collected and for the time frame analyzed, as the causal impact of diseases such as PRRSV is expected to change over time and between different companies or regions if a new PRRSV strain is introduced, for example. This study focused on survivability, not assessing changes in other key performance indicators such as growth rate or feed efficiency. Another limitation of this study is that it is prone to information bias as data recorded in the farm and used in the model could have been recorded incorrectly (e.g., the date of an outbreak with PRRSV in the sow farm could have been reported early or late compared to the actual date). In addition, the information used for characterizing the weekly batches of weaned pigs was provided as average values for the specific weeks over an “X” number of wean events per litter, where the variance within the means was unavailable from the raw data. Finally, other potential diseases not recorded by the system could have influenced the health status of the animals at the time of the weaning.

Despite the limitation, this study demonstrates the importance of applying causal inference models to observational data instead of relying on raw mean comparisons or from regression analyses marginal estimates, as it provided more accurate modeling strategy for analysis of multiple variables with non-linear relationships. The extrapolation of the present results for other field conditions is limited as the interactions between health and productivity are expected to change drastically over time, location, and farms.

## 5 Conclusion

Building causal inference analysis in the veterinary epidemiology realm requires consolidated data containing comprehensive information and appropriate implementation of causal inference methods. This study demonstrated the application of this approach using swine data collected under field conditions by following the guidelines for observational studies and the steps recommended for causal inference analysis.

The goal for applying the causal inference approach, rather than relying only on regression models, was to handle the variation of known background factors related to PRRSV outbreaks in sow farm and downstream nursery mortality, providing accurate estimates between epidemic and non-epidemic lots after reducing the variation of the confounders.

The doubly robust method provided the lowest impact for the two PRRSV lots on mortality, suggesting that the other approaches could not control all confounding effects. This study revealed the application of causal inference models in swine field data, which can be utilized for other pathogens or disease/production-related factors.

## Data Availability

The data analyzed in this study is subject to the following licenses/restrictions: The data are not publicly available due to privacy. Requests to access these datasets should be directed to EM, edison@iastate.edu.
