# Supplementary material for: Measuring the impact of sow farm outbreaks with PRRS virus on the downstream mortality using causal inference methods
Source: Front Vet Sci. 2025 Apr 30;12:1545034. doi: 10.3389/fvets.2025.1545034 (PMC12076741; doi:10.3389/fvets.2025.1545034)
Supplement: Supplementary file 1 [file Table_1.docx]

Appendix 1: Code utilized on the dagitty platform (<https://www.dagitty.net/dags.html>) to create the causal diagram of the impact of PRRS epidemic sow farms on nursery mortality.

dag {

bb="0,0,1,1"

"Avg. parity at farrow" [pos="0.082,0.400"]

"Nursery Mortality" [outcome,pos="0.728,0.418"]

"Other diseases" [latent,pos="0.534,0.867"]

"PRRS epidemic sow farms" [exposure,pos="0.373,0.339"]

"PRRS nursery outbreak" [pos="0.840,0.111"]

"Pre-Wean mortality" [pos="0.517,0.539"]

"Sow source" [pos="0.288,0.146"]

"Stocking weight" [pos="0.542,0.435"]

"Weaning Age" [pos="0.496,0.699"]

Mycoplasma [pos="0.244,0.455"]

Season [pos="0.520,0.161"]

"Avg. parity at farrow" -> "Other diseases" [pos="0.185,0.766"]

"Avg. parity at farrow" -> "PRRS epidemic sow farms" [pos="0.164,0.264"]

"Avg. parity at farrow" -> "Weaning Age" [pos="0.248,0.615"]

"Avg. parity at farrow" -> Mycoplasma

"Other diseases" -> "Nursery Mortality"

"PRRS epidemic sow farms" -> "Nursery Mortality"

"PRRS epidemic sow farms" -> "Pre-Wean mortality"

"PRRS epidemic sow farms" -> "Stocking weight"

"PRRS epidemic sow farms" -> "Weaning Age"

"PRRS nursery outbreak" -> "Nursery Mortality"

"Pre-Wean mortality" -> "Nursery Mortality"

"Sow source" -> "PRRS epidemic sow farms"

"Stocking weight" -> "Nursery Mortality"

"Weaning Age" -> "Nursery Mortality"

Mycoplasma -> "Nursery Mortality" [pos="0.232,0.006"]

Mycoplasma -> "Other diseases"

Mycoplasma -> "PRRS epidemic sow farms" [pos="0.270,0.333"]

Mycoplasma -> "Pre-Wean mortality"

Mycoplasma -> "Stocking weight"

Season -> "Nursery Mortality" [pos="0.657,0.175"]

Season -> "PRRS epidemic sow farms" [pos="0.399,0.150"]

}
